# BIC2, a Cryptochrome Function Inhibitor, Is Involved in the Regulation of ABA Responses in *Arabidopsis*

**DOI:** 10.3390/plants12112220

**Published:** 2023-06-05

**Authors:** Yating Wang, Wei Wang, Qiming Jia, Hainan Tian, Xutong Wang, Yingying Li, Saddam Hussain, Hadia Hussain, Tianya Wang, Shucai Wang

**Affiliations:** 1 Laboratory of Plant Molecular Genetics & Crop Gene Editing, School of Life Sciences, Linyi University, Linyi 276000, China; wangyt814@nenu.edu.cn (Y.W.); wangwei220201@163.com (W.W.); wanxutong0019@163.com (X.W.); 2 Key Laboratory of Molecular Epigenetics of MOE, Northeast Normal University, Changchun 130024, China; jiaqm859@163.com (Q.J.); tianhainan2012@126.com (H.T.); liyy857@nenu.edu.cn (Y.L.); botanistonline@yahoo.com (S.H.); hadiahussain37@outlook.com (H.H.); wangty309@nenu.edu.cn (T.W.)

**Keywords:** BIC2, ABA, CRISPR/Cas9, transcription factor, *Arabidopsis*

## Abstract

The plant hormone ABA (abscisic acid) is able to regulate plant responses to abiotic stresses via regulating the expression of ABA response genes. BIC1 (Blue-light Inhibitor of Cryptochromes 1) and BIC2 have been identified as the inhibitors of plant cryptochrome functions, and are involved in the regulation of plant development and metabolism in *Arabidopsis* . In this study, we report the identification of BIC2 as a regulator of ABA responses in *Arabidopsis* . RT-PCR (Reverse Transcription-Polymerase Chain Reaction) results show that the expression level of *BIC1* remained largely unchanged, but that of *BIC2* increased significantly in response to ABA treatment. Transfection assays in *Arabidopsis* protoplasts show that both BIC1 and BIC2 were mainly localized in the nucleus, and were able to activate the expression of the co-transfected reporter gene. Results in seed germination and seedling greening assays show that ABA sensitivity was increased in the transgenic plants overexpressing *BIC2*, but increased slightly, if any, in the transgenic plants overexpressing *BIC1*. ABA sensitivity was also increased in the *bic2* single mutants in seedling greening assays, but no further increase was observed in the *bic1 bic2* double mutants. On the other hand, in root elongation assays, ABA sensitivity was decreased in the transgenic plants overexpressing *BIC2*, as well as the *bic2* single mutants, but no further decrease was observed in the *bic1 bic2* double mutants. By using qRT-PCR (quantitative RT-PCR), we further examined how BIC2 may regulate ABA responses in *Arabidopsis* , and found that inhibition of ABA on the expression of the ABA receptor genes *PYL4* (*PYR1-Like 4*) and *PYL5* were decreased, but promotion of ABA on the expression of the protein kinase gene *SnRK2.6* (*SNF1-Related Protein Kinases 2.6*) was enhanced in both the *bic1 bic2* double mutants and *35S:BIC2* overexpression transgenic plants. Taken together, our results suggest that BIC2 regulates ABA responses in *Arabidopsis* possibly by affecting the expression of ABA signaling key regulator genes.

## 1. Introduction

CRYs (Cryptochromes) are photolyase-like flavoproteins found in all evolutionary lineages, and plant CRYs are blue light receptors that mediate light regulated plant growth and development and metabolism [1,2,3]. CRYs present as inactive monomers in darkness, but as active homodimers after being photoexcited by blue light to regulate gene expression, and eventually plant growth and development and metabolism in plants [1,2,3,4,5]; whereas, BICs (Blue-light Inhibitor of Cryptochromes) are inhibitors of CRYs. They can bind to CRYs to suppress their blue light-dependent dimerization, photobody formation, phosphorylation, degradation, and physiological activities, therefore affecting CRYs mediated gene expression and photoresponses [2,6,7,8,9].

As mentioned above, CRYs function as homodimers, but BICs are able to act as the competitive inhibitors for CRY2-CRY2 homodimerization, wrapping around the CRY2 to block its photo-oligomerization and photoactivation [10]. It has been shown that the residues W349 and R208 in CRY2 are critical for the formation of both the CRY2-CRY2 and the CRY-BIC complexes. The W349 and R208 are at the interfaces of the CRY2-CRY2 homodimers [11]. The W349 is also able to hydrophobically interacts with the I57 residue in BIC2 in the CRY2-BIC2 heterodimer, and the R208 is able to form a hydrogen bond with E50 residue in BIC2 [12]. On the other hand, it has also been show that there is a negative-feedback regulation loop of CRYs [2,8]. Photoactivation of CRYs enables their interaction with the COP1-SPAs (Constitutive Photomorphogenesis 1-Suppressors of Phytochrome A) complexes, thus suppressing the E3 ubiquitin ligase activity of COP1, resulting in protein accumulation of the HY5 (Elongated Hypocotyl 5) transcription activator. HY5 are then able to associate with chromatins of the *BICs* promoters, activating their transcription. Accumulated BICs, in turn, inhibit CRY2-CRY2 homodimerization [2,8].

BICs are widely distributed in plants and have a conservative trend in the process of biological evolution. For example, similar to *Arabidopsis* *BICs*, the expression of *OsBICs* is blue light-inducible. OsBICs and *Arabidopsis* BICs share high amino acid identities and similarities in the protein conservative domains. They have similar subcellular localization, and are involved in blue light-induced leaf sheath growth [13]. For another example, similar to the functions of *Arabidopsis* BICs, GmBICs are able to regulate blue light-induced stem elongation in soybean [14], whereas SmBICs intact with SmCRY2 to regulate anthocyanins in eggplants [15].

ABA is a key phytohormone in plants, and plays a variety of functions in regulating plant growth and development, such as seed germination and seedling development in multiple dimensions [16,17]. Most importantly, ABA regulates plant responses to various environmental stresses, such as drought, heat, cold, and salinity [18,19]. 

The details of the ABA signaling process in regulating plant abiotic stress responses have been largely elucidated [20,21,22]. In the absence of ABA, group A PP2Cs (Protein Phosphatase 2Cs), the negative regulators of ABA signaling [23,24,25], interact with SnRK2s (Sucrose Non-fermenting 1 (SNF1)-Related Protein Kinases 2s) protein kinases, the positive regulators of ABA signaling, to inhibit their activities [26]. ABA can bind with the receptors composed by PYR1 (Pyrabactin Resistance 1)/PYLs (PYR1-Likes)/RCARs (Regulatory Component of ABA Receptors), enabling their interaction with group A PP2Cs and leading to the release and self-activation of SnRK2s. Activated SnRK2s are then able to activate the downstream ABF/AREB/ABI5 (ABA-responsive element-Binding Protein/ABRE-Binding factor/ABA Insensitive 5)-type bZIP (basic region leucine zipper) transcription factors [23,24,25]. Activation of the ABF/AREB/ABI5-type bZIP transcription factors through signal transduction of PYR/PYL/RCAR receptors, PP2C phosphatases, and SnRK kinases eventually leads to activation or inhibition of ABA response genes and plant responses to abiotic stresses [21,26,27,28]. 

In addition to the ABF/AREB/ABI5-type bZIP transcription factors, members from several different transcription factor families such as the NAC (NAM, ATAF1/2, and CUC) family, the GARP (Golden2, ARR-B, Psr1) family, the R2R3 MYB family, the bHLH (basic Helix-Loop-Helix) family, the WDR (WD40-repeat) family, and some novel transcription factor families, including AITRs (ABA-induced transcription repressors) and ASRs (ABA-induced Serine-rich Repressors), have been show to regulate plant responses to ABA and/or abiotic stresses [29,30,31,32,33,34,35,36,37,38,39]. Most importantly, at least some of the transcription factors were identified from ABA response genes, including unknown function ABA response genes [34,35,37,38,39].

We report here the identification of BIC2 as a regulator of ABA responses in *Arabidopsis* . We found that the expression level of *BIC2* increased greatly in response to ABA treatment, and BIC2 is able to activate reporter gene expression in transfected protoplasts. By examining overexpression transgenic plants and mutants generated for *BICs*, we found that ABA sensitivity is altered in both the *BIC2* overexpression transgenic plants and the *bic2* mutants in seed germination, seedling greening, and root elongation assays. Quantitative RT-PCR analysis show that ABA regulated expression of some ABA signaling component genes was affected in the overexpression transgenic plants and the mutants.

## 2. Results

### 2.1. Expression of BIC2 Is Induced by ABA Treatment

In the process of identifying new regulators of ABA and/or abiotic stress responses by using the strategy described previously [34], we found that *BIC2*, a cryptochrome function inhibitor gene [7], is an ABA response gene, with a FPKM (Reads Per Kilobase per Million mapped reads) of 2.41 in ABA-treated seedlings, compared to 0.95 in control samples. 

**Figure 1:**
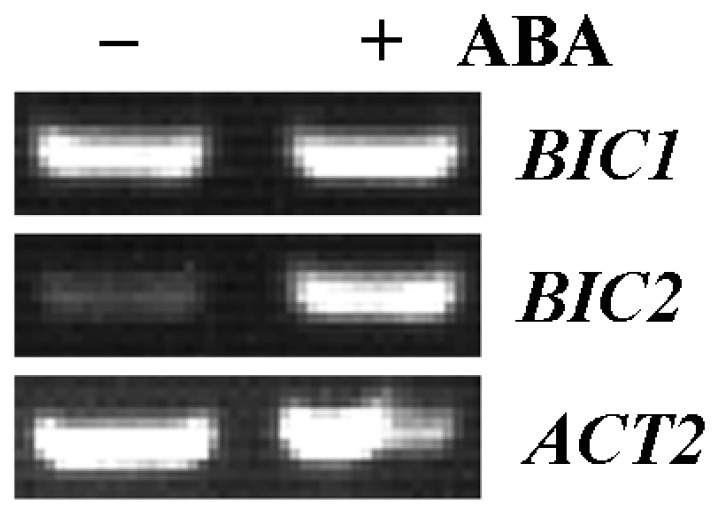
Expression of *BICs* in response to ABA treatment. Fourteen-day-old seedlings of the Col wild type *Arabidopsis* were mock-treated or treated with 50 μM ABA on a shaker at 40 rpm for 4 h in darkness. RNA was then isolated and used for RT-PCR analysis with 30 cycles to examine the expression of *BIC1* and *BIC2*. The expression *ACT2* was used as a control.

To examine if expression of *BIC2* is indeed induced by ABA, we examined the expression of *BIC2* in response to ABA treatment. The Col (Columbia-0) wild type seedlings were treated with ABA or solvent alone as control for a few hours. RNA was then isolated and the expression level of *BIC2* was examined by using RT-PCR. As shown in Figure 1, the expression level of *BIC2* increased dramatically in response to ABA treatment. Considering that BIC1 is closely related to BIC2 [7], we also examined the expression level of *BIC1* in ABA-treated and control seedlings. We found that the expression level of *BIC1* remained largely unchanged in response to ABA treatment (Figure 1). 

### 2.2. BIC1 and BIC2 Activate Reporter Gene Expression in Transfected Protoplasts

It has been shown that BICs are involved in CRYs mediated gene expression [6,7,8,9], and that SmBICs are able to bind directly to the promoter region of eggplant anthocyanin biosynthesis genes in yeast one-hybrid analysis [15]. Considering that *Arabidopsis* BICs appear to be nuclear proteins as indicated by subcellular localization assays in seedlings of the *35S:BIC1-GFP 35S:BIC2-GFP* transgenic plants [7], we therefore examined if BICs may have transcription activities in transfected *Arabidopsis* protoplasts.

**Figure 2:**
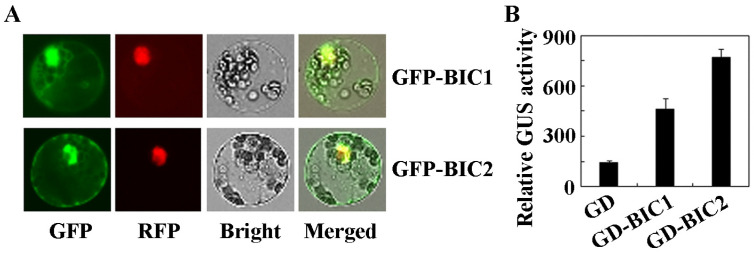
Subcellular localization and transcriptional activities of BIC1 and BIC2. (**A**) Subcellular localization of BIC1 and BIC2. Plasmids of the *GFP-BIC1* and *GFP-BIC2* genes were co-transfected, respectively, with the nuclear indicator gene *NLS-RFP* into *Arabidopsis* protoplasts isolated from the Col wild type plants. The transfected protoplasts were incubated in dark for 18–20 h at room temperature, and then GFP and RFP fluorescence were examined under a fluorescence microscope. (**B**) Transcriptional activities of BIC1 and BIC2. Plasmids of the effector genes *GD*, *GD-BIC1*, and *GD-BIC2* were co-transfected, respectively with the reporter gene *Gal4:GUS* into *Arabidopsis* protoplasts isolated from the Col wild type plants. The transfected protoplasts were incubated in dark for 20–22 h at room temperature, and then GUS activity was assayed by using a microplate reader. Data represent the mean ± SD of three replicates.

We first examined the subcellular localization of BIC1 and BIC2 proteins using *Arabidopsis* protoplast transient transfection assays, to confirm if they are indeed nuclear proteins. Plasmids of *GFP-BIC1* and *GFP-BIC2* effector genes were co-transfected, respectively, with the nucleus indicator gene *NLS-RFP* into *Arabidopsis* protoplasts. The GFP and RFP fluorescence were observed under a fluorescence microscope after the transfected protoplasts were incubated overnight in darkness. As shown in Figure 2A, GFP and RFP fluorescence were mainly observed in the nucleus, indicating that BIC1 and BIC2 were mainly localized in the nucleus.

We then examined the transcriptional activities of BIC1 and BIC2 by using protoplast transfection. Plasmids of the effect gene *GD-BIC1*, *GD-BIC2*, and the control gene *GD* were co-transfected, respectively, with the reporter gene *Gal4:GUS* into *Arabidopsis* protoplasts. The transfected protoplasts were incubated overnight in darkness, and GUS activities were measured by using a microplate reader. As shown in Figure 2B, expression of the reporter gene was activated by co-transfection of the effector gene *GD-BIC1* or *GD-BIC2*, when compared with the co-transfected *GD* control, indicating that BIC1 and BIC2 have transcription activation activities. 

### 2.3. Light-Grown Seedlings of the BICs Overexpression Plants Produced Longer, Whereas the bic1 bic2 Double Mutants Produced Shorter Hypocotyls

**Figure 3:**
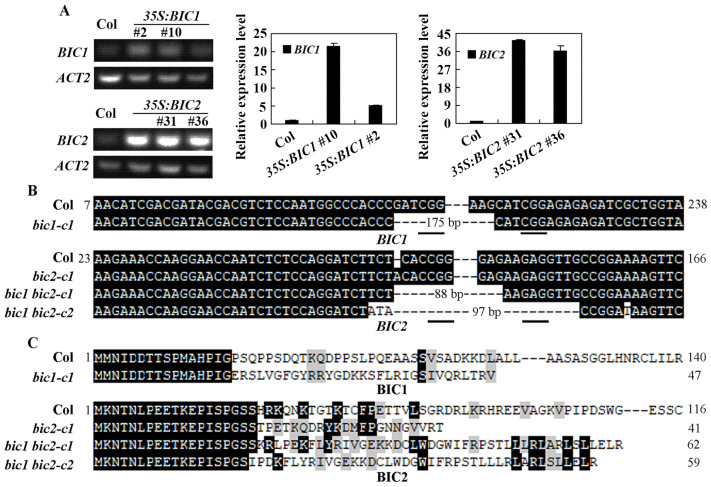
Generation of overexpression transgenic plants and gene edited mutants of *BICs*. (**A**) Expression level of *BICs* in the overexpression transgenic plants. RNA was isolated from 14-day-old seedlings and used for RT-PCR and q-RT-PCR analysis. The expression *ACT2* was used as a control for RT-PCR analysis and an inner control for qRT-PCR analysis. In the qRT-PCR analysis, the expression level of *BIC1*/*BIC2* in the Col was set as 1. Lanes without numbers indicated homozygous lines did not used in the experiments. (**B**) Alignment of the nucleotide sequences of *BIC1* and *BIC2* in Col wild type, the *bic1* and *bic2* single, and the *bic1 bic2* double mutants. The *bic1* and *bic2* single mutants were obtained by transforming the Col wild type plants with *pHEE-BIC1* and *pHEE-BIC2* constructs, respectively, and the *bic1 bic2* double mutants were obtained by transforming the *bic1-1* single mutant plants with the *pHEE-BIC2* construct. DNA was isolated from leaves of T2 or T3 plants and used for PCR amplification of *Cas9* to identify transgene-free mutants and for amplification of *BIC1* and *BIC2* for sequencing to identify homozygous mutants. The sequencing results of the homozygous mutants were aligned with wild type genome sequence of *BIC1* and *BIC2*. Underlines indicate the PAM sites. (**C**) Alignment of the amino acid sequences of BIC1 and BIC2 in the Col wild type, the *bic1* and *bic2* single, and the *bic1 bic2* double mutants. Coding sequences of *BIC1* and *BIC2* in the mutants were subjected to ORFfinder (https://www.ncbi.nlm.nih.gov/orffinder/ (accessed on 1 June 2019)) for ORF analysis, and predicted amino acid sequences were used for alignment with the amino acid sequences of wild type BIC1 and BIC2, respectively. Identical amino acids are shaded in black and similar in gray.

After showing the *BIC2* is an ABA response gene (Figure 1), and BIC1 and BIC2 are nuclear proteins and function as transcription activators (Figure 2), we wanted to examine the roles of BICs in regulating ABA responses in *Arabidopsis* . To do that, overexpression transgenic plants and mutants were generated. The overexpression transgenic plants were generated by transforming the Col wild type plants with the *35S:BIC1* and *35S:BIC2* construct, respectively, and selecting homozygous transgenic plants in the T3 generation. Two homozygous of overexpression transgenic plant lines for *BIC1* and *BIC2*, respectively, i.e., *35S:BIC1* #2 and #10, and *35S:BIC2* #31 and #36 (Figure 3A), were used for the experiments. RT-PCR results show that the expression level of *BIC1*/*BIC2* was increased in the transgenic lines. Quantitative RT-PCR results show that the expression level of *BIC1* increased ~5 and ~20 folds in the two *35S:BIC1* transgenic lines, respectively, whereas that of *BIC2* increased ~40 folds in the two *35S:BIC2* transgenic lines, respectively.

Single mutants were isolated from T-DNA insertion lines, and generated by using CRISPR/Cas9 to edit *BIC1* and *BIC2*, respectively, in the Col wild type plants. T-DNA insertion lines GK-014D08 and SM-3-21731 were obtained from ABRC, and used to isolate the *bic1-1* and the *bic2-1* single mutants, respectively, which are the mutants used in a previously study [7]. In addition, we generated gene-edited single mutants for *BIC1* and *BIC2*, respectively, by using CRISPR/Cas9 gene editing in the Col wild type plants. Two target sequences for each of the two genes were selected and used to generate CRISPR/Cas9 constructs for plant transformation. The transgene-free mutants were isolated in T2 or T3 generations by isolated Cas9-free plants and sequencing the *BIC1* and *BIC2* genome sequences. Two gene edited single mutants, i.e., *bic1-c1* and *bic2-c1*, were obtained and used for the experiments. Double mutants were generated by using CRISPR/Cas9 to edit *BIC2* in the *bic1-1* mutant plants, and two double mutants, i.e., *bic1 bic2-c1* and *-c2*, were obtained and used for the experiments. 

In the *bic1-c1* mutant, both of the two target sequences in *BIC1* were edited, and a 175 bp deletion occurred between the two target sequences. In the *bic2-c1* mutant, however, only one target sequence in *BIC2* was edited, and a single nucleotide was inserted in the first target sequence (Figure 3B). In the *bic1 bic2* double mutants, both of the target sequences in *BIC2* were edited. An 88 bp deletion occurred between the two target sequences in the *bic1 bic2-c1* mutant, and a 97 bp deletion together with 4 nucleotide substitutions occurred in the *bic1 bic2-c2* mutant (Figure 3B). All of the mutation resulted in amino acid substitutions and premature stops for BIC1 and BIC2, respectively, in the mutants obtained (Figure 3C). 

**Figure 4:**
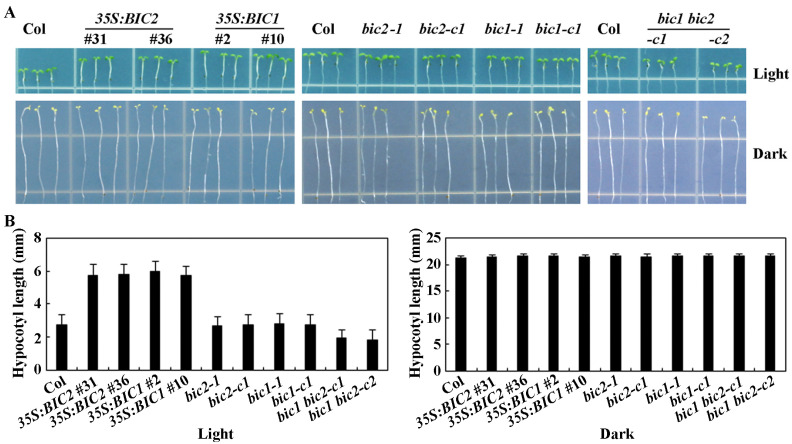
Hypocotyl length of light- and dark-grown seedlings of the Col wild type, and the overexpression transgenic plants and mutants of *BICs*. (**A**) Images of representative light- and dark-grown seedlings. Sterilized seeds of the Col wild type, the *35S:BIC1* and *35S:BIC2* transgenic plants, the *bic1* and *bic2* single, and the *bic1 bic2* double mutants were germinated and grown on vertically placed ½ MS plates solidified with 1.5% agar in a growth room under light for 5 days or dark condition for 7 days, then photographed using a digital camera. (**B**) Hypocotyl length of light- and dark-grown seedlings. Hypocotyl length of 5-day-old light-grown seedlings or 7-day-old dark-grown seedlings on vertically placed ½ MS plates was measured. Data represent the mean ± SD of 15–18 seedlings.

By growing all the overexpression transgenic plants and the mutants generated together with the Col wild type in ½ MS plates in light and dark conditions, we observed hypocotyl elongation. We found that under the dark-grown conditions, all the seedlings including the Col wild type plants, the *35S:BIC1* and *35S:BIC2* transgenic plants, the *bic1* and *bic2* single, and the *bic1 bic2* double mutants produced hypocotyls with similar length (Figure 4A). However, under light-grown condition, seedlings of the *35S:BIC1* and *35S:BIC2* transgenic plant produced longer hypocotyls, whereas seedlings of the *bic1 bic2* double mutants produced shorter hypocotyls compared with the Col wild type seedlings (Figure 4A). 

Quantitative results show that the hypocotyl length of the light-grown seedlings of the Col wild type was ~3 mm, that of the *35S:BIC1* and *35S:BIC2* transgenic plant seedlings was ~6 mm, that of the *bic1* and *bic2* single mutant seedlings was similar to that of the Col wild type seedlings, but that of the *bic1 bic2* double mutant seedlings was only ~2 mm. On the other hand, dark-grown seedlings of the all the plants produced hypocotyls at ~21 mm (Figure 4B). These results are comparable to the results obtained under blue light-grown and dark-grown conditions [7].

### 2.4. ABA Sensitivity Is Altered in Both the 35S:BIC2 Transgenic Plants and the bic2 Mutants

The expression level of *BIC2* was increased in response to ABA treatment (Figure 1), suggesting that BIC2 may be involved in the regulation of ABA responses. We therefore compared ABA responses of the overexpression transgenic plants and the mutants of *BICs* obtained with the Col wild type plants, by using ABA inhibited seed germination, seedling greening, and root elongation assays. 

**Figure 5:**
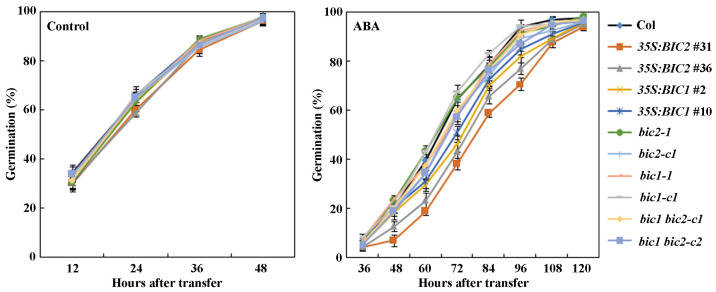
Effects of ABA on seed germination of the Col wild type, and the overexpression transgenic plants and mutants of *BICs* in response to ABA treatment. Sterilized seeds of the Col wild type, the *35S:BIC1* and *35S:BIC2* transgenic plants, the *bic1* and *bic2* single, and the *bic1 bic2* double mutants were plated on 1.0% agar solidified ½ MS plates with or without 1 μM ABA. The plates were kept at 4 °C for 2 days in darkness, and then transferred to a growth room. The number of seeds germinated was counted every 12 h started 12 h after the transfer for control plates and 36 h after for ABA treated plates, and percentage of germination was calculated. Data represent the mean ± SD of four replicates.

In the seed germination assays, all the seeds in the control plates, including seeds of Col wild type, the *35S:BIC1* and *35S:BIC2* transgenic plants, the *bic1* and *bic2* single, and the *bic1 bic2* double mutants germinated 48 h after the plates were transferred to a growth room (Figure 5). 

However, on the ABA-containing plates, lower germination rate was observed for seeds of the *35S:BIC1* and *35S:BIC2* transgenic plants at most of the time points examined, as compared to seeds of the Col wild type plants. We also noted that seeds of the *35S:BIC2* transgenic plant have a relative lower germination rate compared with seeds of the *35S:BIC1* transgenic plants. On the other hand, no difference was observed for between seeds of the Col wild type and seeds of the *bic1* and *bic2* single and the *bic1 bic2* double mutants (Figure 5). Nevertheless, these results show that ABA sensitivity is increased in the *35S:BIC1* and *35S:BIC2* transgenic plants.

**Figure 6:**
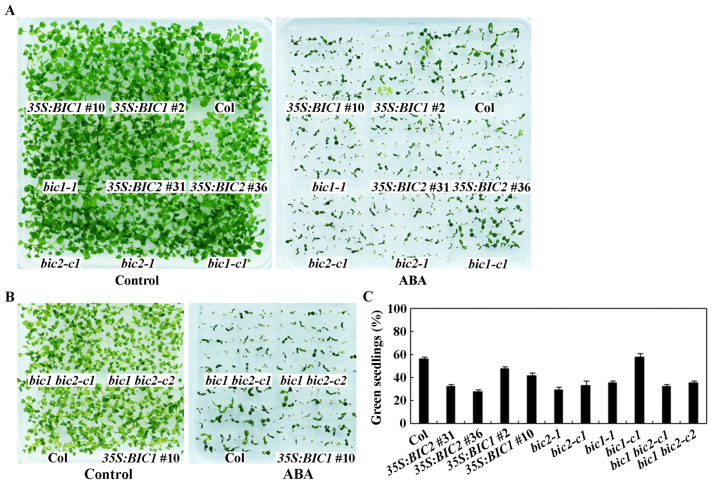
Seedling greening and percentage of green seedlings of the Col wild type, the overexpression transgenic plants, and mutants of *BICs* in response to ABA treatment. (**A**) Representative images of seedling of the Col wild type, and the overexpression transgenic plants and single mutants of *BICs* in control and ABA plates. Sterilized seeds of the Col wild type, the *35S:BIC1* and *35S:BIC2* transgenic plants, and the *bic1* and *bic2* single mutants were plated on 1.0% agar solidified ½ MS plates with or without 1 µM ABA. The plates were kept at 4 °C for 2 days in darkness, and then transferred to a growth room. Pictures were taken 14 days after the transfer. (**B**) Representative images of seedling of the Col wild type, and the overexpression transgenic plants and double mutants of *BICs* in control and ABA plates. Sterilized seeds of the Col wild type, the *35S:BIC1* #10 transgenic plants, and the *bic1 bic2* double mutants were plated on 1.0% agar solidified ½ MS plates with or without 1 µM ABA. The plates were kept at 4 °C for 2 days in darkness, and then transferred to a growth room. Pictures were taken 13 days after the transfer. (**C**) Percentage of green seedlings of the Col wild type, and the overexpression transgenic plants, single and double mutants of *BICs* in ABA plates. Seedlings with green cotyledons were counted 14 days after the transfer and percentage of green seedlings was calculated. Data represent the mean ± SD of four replicates.

In the seedling greening assays, clearly increased ABA sensitivity was also observed for the *35S:BIC2* transgenic plants, but only a slight increase was observed for the *35S:BIC1* transgenic plants (Figure 6A). To our surprise, we found that ABA sensitivity also increased in the *bic2* single mutants (Figure 6A), but did not further increase in the *bic1 bic2* double mutants (Figure 6B). However, different ABA responses were observed for the two *bic1* single mutants, increased ABA sensitivity was observed in the *bic1-1* mutant, but that of the *bic1-c1* mutant was largely similar to the Col wild type plants (Figure 6B).

Quantitative results show that the percentage of greening seedlings reduced greatly in the *35S:BIC2* transgenic plants, the *bic2* and the *bic1-1* single, and the *bic1 bic2* double mutants, but only reduced slightly in the *35S:BIC1* transgenic plants. No difference was observed between the *bic1-c1* single mutant and the Col wild type plants (Figure 6C).

**Figure 7:**
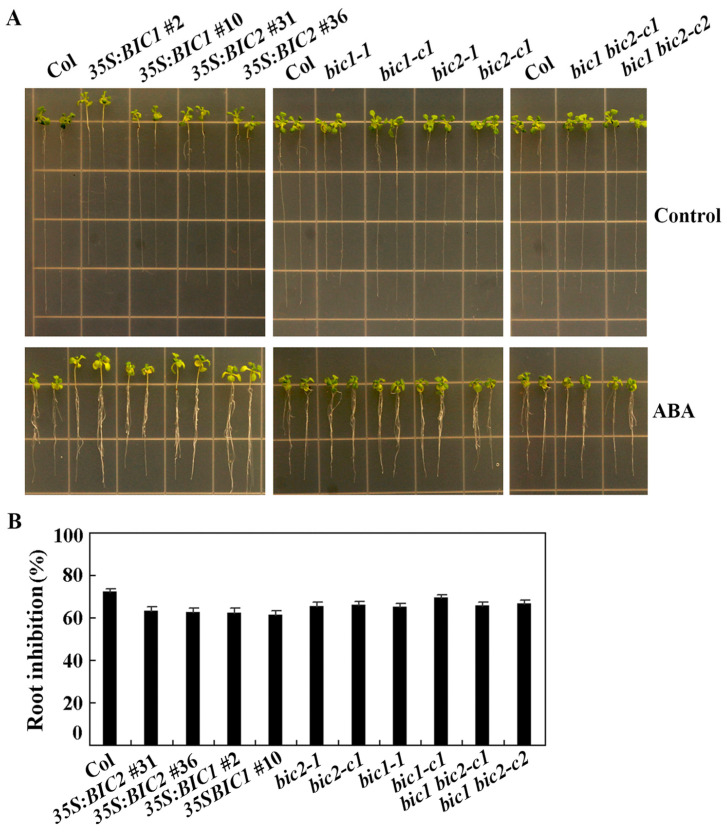
Root elongation of the Col wild type, the overexpression transgenic plants and mutants of *BICs* in response to ABA treatment. (**A**) Images of representative grown on control and ABA plates seedlings. Sterilized seeds of the Col wild type, the *35S:BIC1* and *35S:BIC2* transgenic plants, the *bic1* and *bic2* single, and the *bic1 bic2* double mutants were germinated and grown on vertically placed ½ MS plates solidified with 1.5% agar in a growth room for 5 days. The seedlings were then transferred to control plates and plates containing 10 μM ABA and grown for 8 more days, then pictures were taken by using a digital camera. (**B**) Percentage of root inhibition by ABA. Length of new elongated roots was measured, and percentage of inhibition was calculated. Data represent the mean ± SD of 20–22 seedlings.

In the root elongation assays, ABA inhibited root elongation of all the plants. However, decreased ABA sensitivity was observed for the *35S:BIC2* and the *35S:BIC1* transgenic plants, the *bic1-1* and *bic2* single mutants, and the *bic1 bic2* double mutants (Figure 7A). Quantitative results show that ABA sensitivity in the *bic1-c1* single mutant was largely similar to the Col wild type plants, and no further changes were observed in the *bic1 bic2* double mutants when compared to the *bic1-1* and *bic2* single mutants (Figure 7B).

### 2.5. The Expression of Some ABA Signaling Core Regulator Genes Were Affected in the bic1 bic2 Double Mutants and the 35S:BIC2 Transgenic Plants

**Figure 8:**
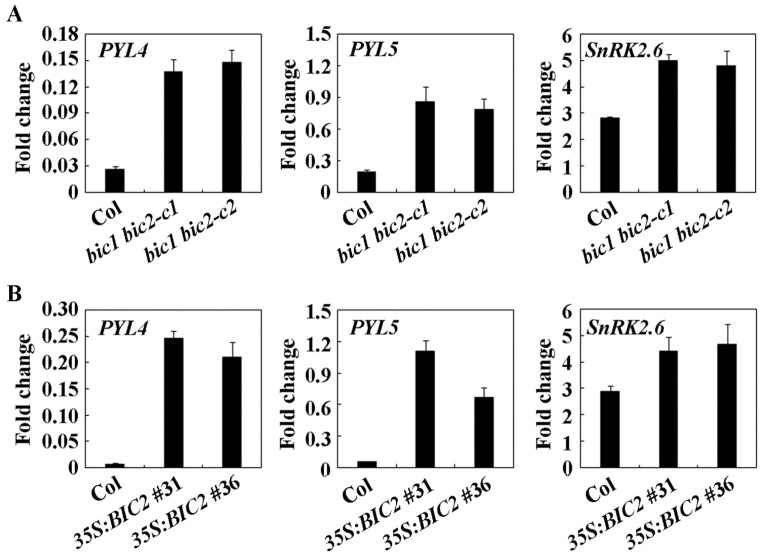
Fold changes of the expression of *PYL4*, *PYL5*, and *SnRK2.6* in response to ABA in the Col wild type, the *bic1 bic2* double mutants, and the *35S:BIC2* transgenic plants. (**A**) Fold changes of the expression of *PYL4*, *PYL5*, and *SnRK2.6* in response to ABA in the Col wild type and the *bic1 bic2* double mutants. (**B**) Fold changes of the expression of *PYL4*, *PYL5*, and *SnRK2.6* in response to ABA in the Col wild type and the *35S:BIC2* transgenic plants. Fourteen-day-old seedlings of the Col wild type, the *bic1 bic2* double mutants, and the *35S:BIC2* transgenic plants were treated with 50 μM ABA or mock treated on a shaker at 40 rpm for 4 h in darkness. RNA was isolated and used for qRT-PCR analysis. Expression of *ACT2* was used as an inner reference gene, and fold changes were calculated by comparing the transcript level of the corresponding genes in ABA treated and control seedlings. Data represent the mean ± SD of three replicates.

Even though ABA response was not consistent in the two *bic1* mutants, our above results show that that ABA sensitivity was altered in *35S:BIC2* transgenic plants, and the *bic2* single and *bic1 bic2* double mutants compared to the Col wild type (Figure 5, Figure 6 and Figure 7). These results indicate that BIC2 is involved in the regulation of ABA responses in *Arabidopsis* . To examine how BIC2 may regulate ABA responses in *Arabidopsis* , we wanted to examine if the expression of the ABA signaling core regulator genes was affected by BIC2. Considering that similar ABA sensitivity was observed in the *bic2* single and *bic1 bic2* double mutants (Figure 6), and the *bic1 bic2* mutants showed light-mediated growth phenotypes (Figure 4), we used the *bic1 bic2* double mutants rather than the *bic2* single mutants for the experiment. As shown in Figure 8A, inhibition of ABA on the expression of *PYL* genes *PYL4* and *PYL5* were decreased, whereas promotion of ABA on the expression of the *SnRK2* gene *SnRK2.6* was enhanced in the *bic1 bic2* mutants. We then examined the expression of the ABA signaling core regulator genes in the *35S:BIC2* transgenic plants, and the results were similar to that obtained in the *bic1 bic2* mutants; i.e., inhibition of ABA on the expression of *PYL4* and *PYL5* were decreased, whereas promotion of ABA on the expression of the *SnRK2.6* was enhanced in the *35S:BIC2* transgenic plants (Figure 8B). These results suggest that BIC2 may regulate ABA responses in *Arabidopsis* by regulating the expression of ABA signaling core regulator genes.

## 3. Discussion

BIC1 and BIC2 can bind to CRYs to suppress their activities, thereby affecting CRYs mediated gene expression and photoresponses, eventually light mediated plant growth and development, and metabolisms [2,6,7,8,9]. In this study, we provide evidences that BIC2 is also involved in the regulation of ABA responses in *Arabidopsis* . 

First, we identified *BIC2* as an ABA response gene, and RT-PCR result show that the expression level of *BIC2* increased dramatically in response to ABA treatment (Figure 1). Second, by generating transgenic overexpression plants and mutants for *BIC2* gene, and using them for ABA sensitivity assays, we found that the *35S:BIC2* transgenic plants showed altered sensitivity to ABA treatment in seed germination, seedling greening, and root elongation assays (Figure 5, Figure 6 and Figure 7), and the *bic2* single mutants showed altered ABA sensitivity too in seedlings greening and root elongation assays. Third, the ABA-regulated expression of some ABA signaling core regulator genes including *PYL4*, *PYL5* and *SnRK 2.6* was changed in the *bic1 bic2* double mutants and the *35S:BIC2* transgenic plants (Figure 8). 

The *bic1 bic2* double mutants, but not the *bic2* single mutants, produced shorter hypocotyl under light-grown condition (Figure 4), a result similar to the observations under blue light condition [7], suggesting that the *bic1 bic2* double mutants we generated should be loss-of-function mutants for both *BIC1* and *BIC2* genes. In addition, even though no difference in ABA sensitivity was observed for the *bic2* single and the *bic1 bic2* double mutants (Figure 6 and Figure 7), slight changes in ABA sensitivity were observed for the *35S:BIC1* transgenic plants (Figure 5 and Figure 7), suggesting that BIC1 may also have a role in regulating ABA responses. Therefore, the *bic1 bic2* double mutants, rather than the *bic2* single mutants, were used to examine the expression of ABA signaling core regulator genes in our experiments. However, it may be of interest to examine the expression of ABA signaling core regulator genes in the *bic2* single mutants to see if BIC1 has a role, and if BIC1 and BIC2 may have redundant functions in regulating ABA responses in *Arabidopsis* .

On the other hand, previous experiments have shown that the expression of *BIC1* was undetectable in the *bic1-1* T-DNA insertion mutant, indicating that *bic1-1* is a loss-of-function mutant [7]. Our *bic1 bic2* double mutants generated in the *bic1-1* mutant showed shorter hypocotyl phenotype under light-grown condition, a phenotype similar to the T-DNA insertion *bic1 bic2* double mutants under blue light-grown condition [7], further confirming that the T-DNA insertion in *BIC1* resulted in loss-of-function mutation. However, ABA sensitivity was changed greatly in the *bic1-1* mutant, but not the *bic1-c1* single mutant in seedling greening assays (Figure 6 and Figure 7). Considering that gene editing of *BIC1* in the *bic1-c1* mutant resulted in a few amino acid substitutions and premature stops for BIC1, and produced a truncated BIC1 protein with only the first 16 amino acids remained unchanged, it is possible that the N-terminal of BIC1 is critical for its function in regulating ABA responses. However, considering that insertion of the T-DNA in *BIC1* in the *bic1-1* mutant may also result in a C-terminal truncated BIC1 protein, we could not rule out the possibility that other genes may be also affected in the gene edited *bic1-c1* mutant. On the other hand, similar ABA sensitivity was observed in the *bic1-1* and *bic2* single and the *bic1 bic2* double mutants in seedling greening assays (Figure 6), even though we could not rule out the possibility that BIC1 and BIC2 may have redundant functions in regulating ABA responses as they are closely related proteins. It is possible that BIC1 and BIC2 may function in parallel pathways to regulate ABA response in *Arabidopsis* . It is worthwhile to generate other *bic1* mutants to further examine if that is the case. 

The protoplast transfection assays indicated that BIC2 is a nuclear protein and is able to activate the expression of the co-transfected reporter gene (Figure 2B), suggesting that BIC2 may function as a transcription activator. However, similar changes of some ABA signaling core regulator genes were observed in the *bic1 bic2* double mutants and the *35S:BIC2* transgenic plants (Figure 7), suggesting that overexpressing and knockout of *BIC2* have similar effects on the expression of the ABA signaling core regulator genes. Thus, it is very unlikely that BIC2 may directly regulate the expression of these ABA signaling core regulator genes. It is possible that protein homeostasis of BIC2 may be important for its functions in regulating ABA responses, and accumulation of more or less BIC2 proteins resulted in similar changes in expression of ABA signaling core regulator genes, therefore, similar ABA responses in Arabidopsis.

## 4. Materials and Methods

### 4.1. Plant Materials and Growth Conditions

All the mutants and transgenic plants are in the Col background, and Col *Arabidopsis* was used for plant transformation and protoplast isolation. The T-DNA insertion lines GK-014D08 and SM-3-21731 were obtained from the *Arabidopsis* Biological Resource Center (Ohio State University, Columbus, OH, USA), and used to identify the homozygous *bic1-1* and *bic2-1* mutants, respectively. The *bic1-c1* and *bic2-c1* single and the *bic1 bic2-c1* and *-c2* double mutants were generated by using CRISPR/Cas9 gene editing.

For ABA treatment and gene expression analysis, seeds of the Col, the *35S:BIC1* and *35S:BIC2* transgenic plants, the *bic1* and *bic2* single, and the *bic1 bic2* double mutants were surface sterilized and sown on plates containing ½ MS salts solidified with 1% or 1.5% agar. The plates were kept at 4 °C in darkness for 2 days, and then transferred into a plant growth room. To generate plants for plant transformation and protoplast isolation, seeds of the Col wild type and *bic1-1* mutant plants were germinated and grown in soil pots. The conditions of the growth room were described previously [37].

### 4.2. ABA Treatment, RNA Isolation, RT-PCR and Quantitative RT-PCR (qRT-PCR)

To examine the expression of *BICs* in response to ABA treatment in the Col wild type seedlings, and the expression of ABA signaling component genes in seedlings of the Col wild type, the *bic1 bic2* double mutants, and the *35S:BIC2* transgenic plants, 14-day-old seedlings were treated with 50 μM ABA in darkness on a shaker at 40 rpm for 4 h and collected and frozen in liquid N_2_ used for RNA isolation. Seedlings treated with the solvent methanol were used as a control. 

Total RNA was isolated by using an EasyPure plant RNA kit (Transgen Biotech, Beijing, China), and 2 μg of the total RNA isolated was subjected to first-strand cDNA synthesis by using an EasyScript First-strand DNA Synthesis Super Mix (TransGen Biotech). Synthesized cDNA was used as a template for RT-PCR or qRT-PCR analysis. The expression of *ACT2* (*ACTIN2*) was used as an inner control. The primers used for qRT-PCR analysis of ABA signaling component genes were as described previously [33,34].

### 4.3. Constructs

The reporter construct *Gal4:GUS*, the effector construct *GD*, and the nuclear indicator construct *NLS-RFP* used for protoplast transfection were as described previously [40,41]. 

To generate constructs for protoplast transfection, the full-length open reading frame (ORF) of *BIC1* and *BIC2* were amplified by RT-PCR using RNA isolated from 12-day-old Col seedlings as template, and cloned in frame with an N-terminal GD and GFP tag into the *pUC19* vector under the control of the double *CaMV 35S* promoter. 

To generate *35S:BIC1* and *35S:BIC2* constructs for plant transformation, the full-length ORF of *BIC1* and *BIC2* were amplified by RT-PCR, cloned in frame with an N-terminal HA tag into the *pUC19* vector under the control of the double *CaMV 35S* promoter, and then digested with proper enzymes and subcloned into the binary vector *pPZP211* [42]. 

To generate CRISPR/Cas9 constructs for genome editing of *BIC1* and *BIC2*, exon sequences of *BIC1* and *BIC2* were subjected to CRISPRscan (http://www.crisprscan.org/?page=sequence (accessed on 1 June 2019) to identify appropriate target sequences. Selected target sequences were then evaluated with Cas-OFFinder (http://www.rgenome.net/cas-offinder/ (accessed on 1 June 2019). Two target sequences were selected for each of the *BICs* gene. The target sequences used for editing *BIC1* were 5′ -TCTCCAATGGCCCACCCGAT(CGG)-3′ and 5′ -GAGAGAGGTTAAAGAAGCAT(CGG)-3′; for *BIC2* were 5′ -TCTCTCCAGGATCTTCTCAC(CGG)-3′ and 5′ -GCTAAAGAGACATAGAGAAG(AGG)-3′. The *pHEE401E* vector was used to generate CRISPR/Cas9 construct by following the procedures described previously [43].

### 4.4. Plant Transformation and Over-Expression Transgenic Plants and Mutants Isolation

The Col wild type and the *bic1-1* mutant plants about 5 weeks old with several mature flowers were transformed with the *35:BIC1* and *35:BIC2* construct in *pPZP211* or CRISPR/Cas9 gene editing constructs by using floral dip method via *Agrobacterium tumefaciens* strain *GV3101* mediated transformation [44]. Homozygous overexpression transgenic plants and Cas9-free homozygous mutants were selected by following the produces as described previously [45,46].

### 4.5. Plasmid DNA Isolation, Protoplasts Isolation and Transfection

Plasmid DNA of the reporter and the effector were isolated by using the GoldHi Endo Free Plasmid Maxi Kit (CWBIO, Taizhou, China) following the manufacture’s procedures, and the concentration of the plasmid DNA was measured by using a NanoDrop (ThermoFisher, Waltham, MA, USA). 

Protoplasts were isolated from leaves of ~4-week-old Col wild type plants and transfected as described previously [34,47,48,49,50]. In brief, for protein subcellular location assay, plasmids of *GFP-BIC1* and *GFP-BIC2* effector constructs were co-transfected, respectively, with the *NLS-RFP* nuclear indicator construct into the protoplasts isolated. For transcriptional activity assays, plasmids of *GD*, *GD-BIC1*, and *GD-BIC2* effect constructs were co-transfected, respectively, with the *Gal4:GUS* reporter construct into the protoplasts isolated. The transfected protoplasts were incubated at room temperature for 20–22 h under darkness, and then GFP and RFP florescence were examined under a fluorescence microscope (Olympus, Tokyo, Japan), whereas GUS activities were measured by using a Synergy HT fluorescence microplate reader (BioTEK, Charlotte, VT, USA). The experiments were repeated at least twice.

### 4.6. Hypocotyl Elongation Assays

For hypocotyl elongation assay, sterilized seeds of the Col wild type, the *35S:BIC1* and *35S:BIC2* transgenic plants, the *bic1* and *bic2* single, and the *bic1 bic2* double mutants were germinated and grown on vertically placed ½ MS plates solidified with 1.5% agar in a growth room under light or dark conditions. In all the assays, 15–18 seedlings for each genotype were used, and the experiments were repeated at least twice.

### 4.7. ABA Sensitivity Assays

ABA inhibited seed germination and seedling greening were assayed as described previously [33,34,37,51]. Briefly, surface sterilized seeds of the Col wild type, the *35S:BIC1* and *35S:BIC2* transgenic plants, the *bic1* and *bic2* single, and the *bic1 bic2* double mutants were sown on plates with ½ MS salts solidified with 1.0% agar in the presence or absence of 1 µM ABA, kept at 4 °C in darkness for 2 days, and then transferred to a growth room. Germinated seeds were counted every 12 h after the transfer from 12h and 36h, respectively, for the control and the ABA treated plates. Pictures were taken 13 and 14 days after the transfer, and seedlings with green cotyledons were counted. The experiments were repeated at least twice.

Root elongation assays were performed as described previously [33]. Briefly, sterilized seeds of the Col wild type, the *35S:BIC1* and *35S:BIC2* transgenic plants, the *bic1* and *bic2* single, and the *bic1 bic2* double mutants were sown on ½ MS plates solidified with 1.5% agar, kept at 4 °C in darkness for 2 days, transferred to a growth room and grown vertically for 5 days, and then transferred to control plates and plates containing 10 μM ABA grown vertically for 8 more days. The length of new elongated roots was measured, and percentage of inhibition was calculated.

## 5. Conclusions

Our results show that *BIC2* is an ABA responsive gene, and BIC1 and BIC2 act as a transcription activator and are involved in the regulating of ABA response in *Arabidopsis* possible by affecting the expression of some ABA signaling core regulator genes.

## Data Availability

All data are presented in the manuscript.
